# A Rare Case of Cutaneous Metastases From Lung Cancer

**DOI:** 10.7759/cureus.119537

**Published:** 2026-11-12

**Authors:** Srinivas Vinjamuri, Zarin Tasnim Parisa, Md Al Amin Sarkar, F M Tanvir Alam

**Affiliations:** 1 Geriatrics, Medway NHS Foundation Trust, Gillingham, GBR; 2 Internal Medicine, Medway NHS Foundation Trust, Gillingham, GBR; 3 Acute Medicine/General Internal Medicine, Medway NHS Foundation Trust, Gillingham, GBR

**Keywords:** advanced malignant tumor, cutaneous malignancy, malignant tumor dressing, metastatic non-small cell lung cancer, squamous cell carcinoma

## Abstract

We present an uncommon case of cutaneous metastasis from non-small cell lung cancer in an 88-year-old woman without systemic symptoms. The initial presentation of an ulceronodular lesion on the right arm led to the diagnosis of a metastatic lung primary, which was confirmed by histopathology and immunohistochemistry. This case illustrates the diagnostic importance of considering related internal malignancies when evaluating atypical skin lesions in elderly patients, even without systemic symptoms.

## Introduction

Although lung cancer is one of the more frequent primary malignancies that can spread to the skin, it is uncommon for cutaneous lesions to be the initial manifestation of the disease [1]. It is the third most common malignancy worldwide [2]. Lung cancer commonly metastasizes to the brain, bone, liver, and adrenal glands, whereas cutaneous involvement is comparatively uncommon. Published estimates of the frequency of skin metastases vary across clinical and pathological series and should be interpreted in light of differences in case ascertainment and tumor populations [3-5]. Although adenocarcinoma is frequently reported among lung primaries producing skin metastases, squamous cell carcinoma is also represented; the relative proportions vary between studies. Cutaneous metastases can arise before, at the time of, or after recognition of the primary lung malignancy, making the interval between pulmonary disease and skin presentation variable rather than predictable.

Skin metastases often appear as firm dermal or subcutaneous nodules, sometimes progressing to ulceration, necrosis, pain, or bleeding. Thoracic and other proximal sites are more typical than the distal upper extremities. A rapidly enlarging forearm or thumb lesion may therefore initially suggest a primary cutaneous squamous cell carcinoma, a keratoacanthoma-type lesion, or an infective process rather than an occult pulmonary malignancy. Cutaneous metastasis generally indicates advanced disease and has important prognostic and palliative implications [4-7].

We report a patient in whom progressive forearm and thumb lesions led to the diagnosis of lung squamous cell carcinoma despite the absence of reported systemic symptoms. The principal clinical lesson is the need for timely tissue diagnosis and careful clinicopathological correlation in an atypical skin tumor.

## Case presentation

We present the case of an 88-year-old woman with stage 3 chronic kidney disease, hypertension, and osteoarthritis who noticed a small nodular lesion on her right forearm. Over approximately five months, the lesion enlarged rapidly and became ulcerated [4], painful, and prone to contact bleeding (Figures 1-3). Approximately three months after the initial forearm lesion appeared, a separate nodule developed on the right thumb and subsequently ulcerated (Figures 4-6). The clinical photographs demonstrate progressive exophytic and necrotic-appearing lesions at both sites.

**Figure 1:**
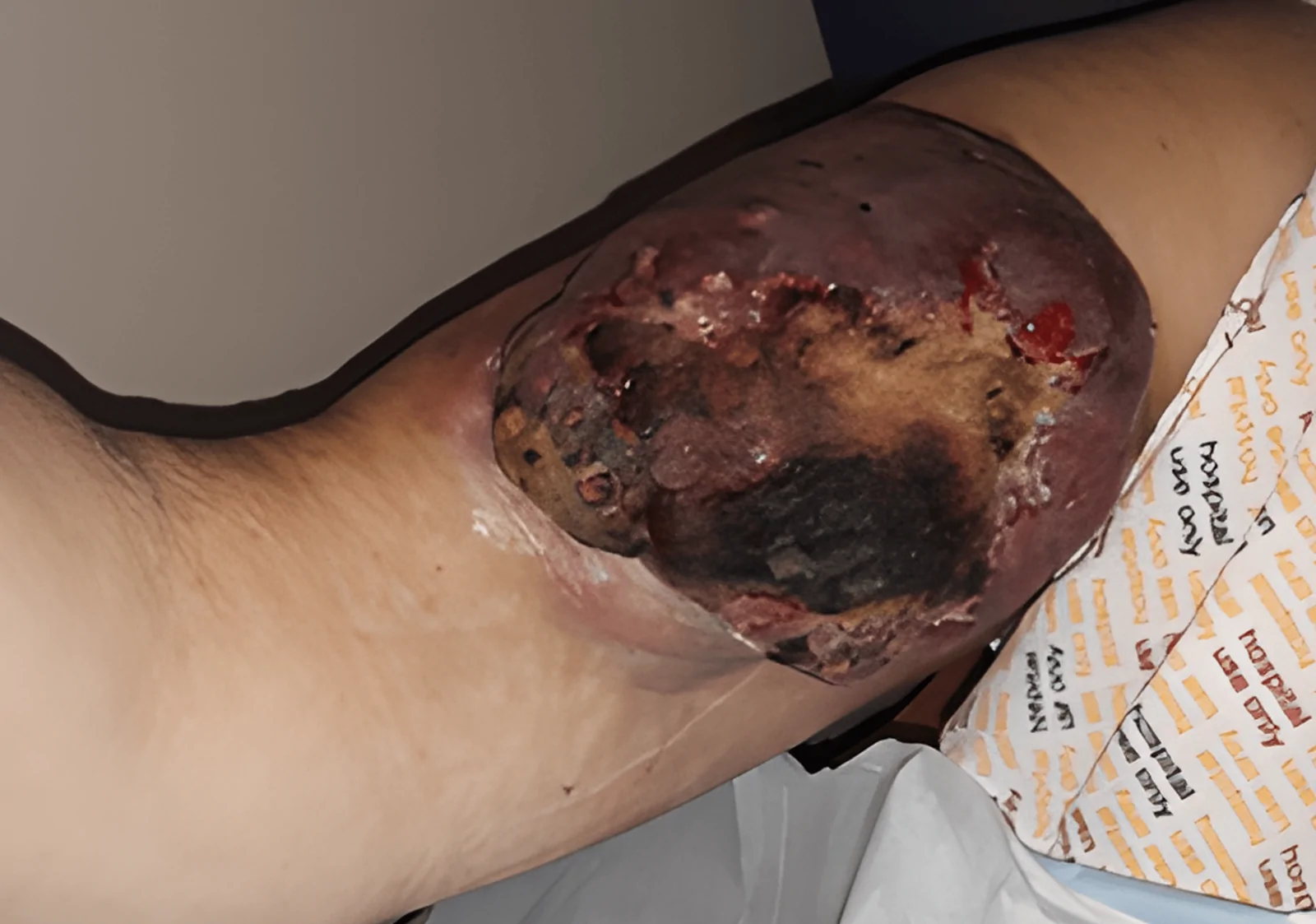
Right forearm: Close-up photograph of the enlarging exophytic ulcerated lesion with areas of surface necrosis and hemorrhage.

**Figure 2:**
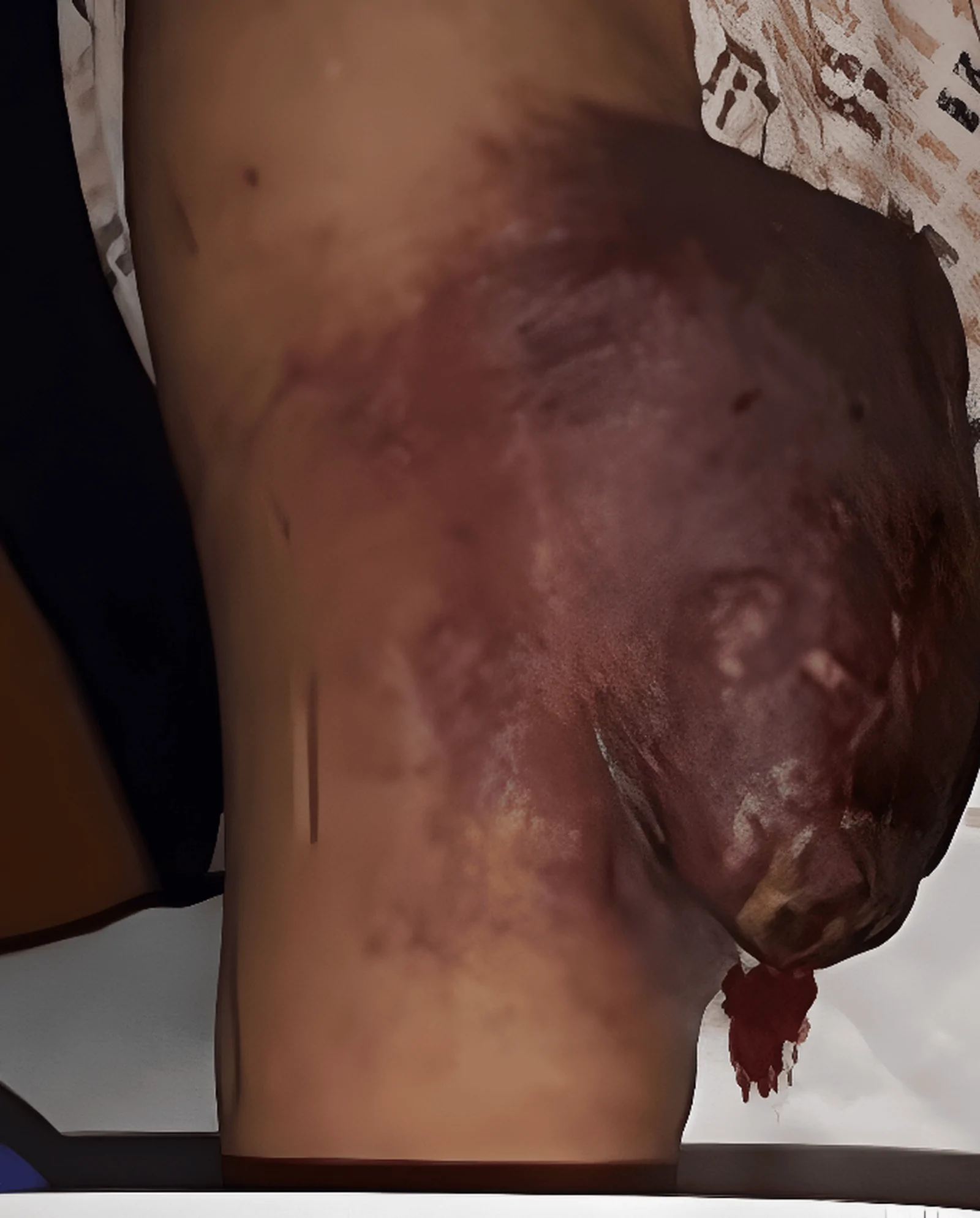
Right forearm: Further view demonstrating the extensive protruding lesion and surrounding skin changes.

**Figure 3:**
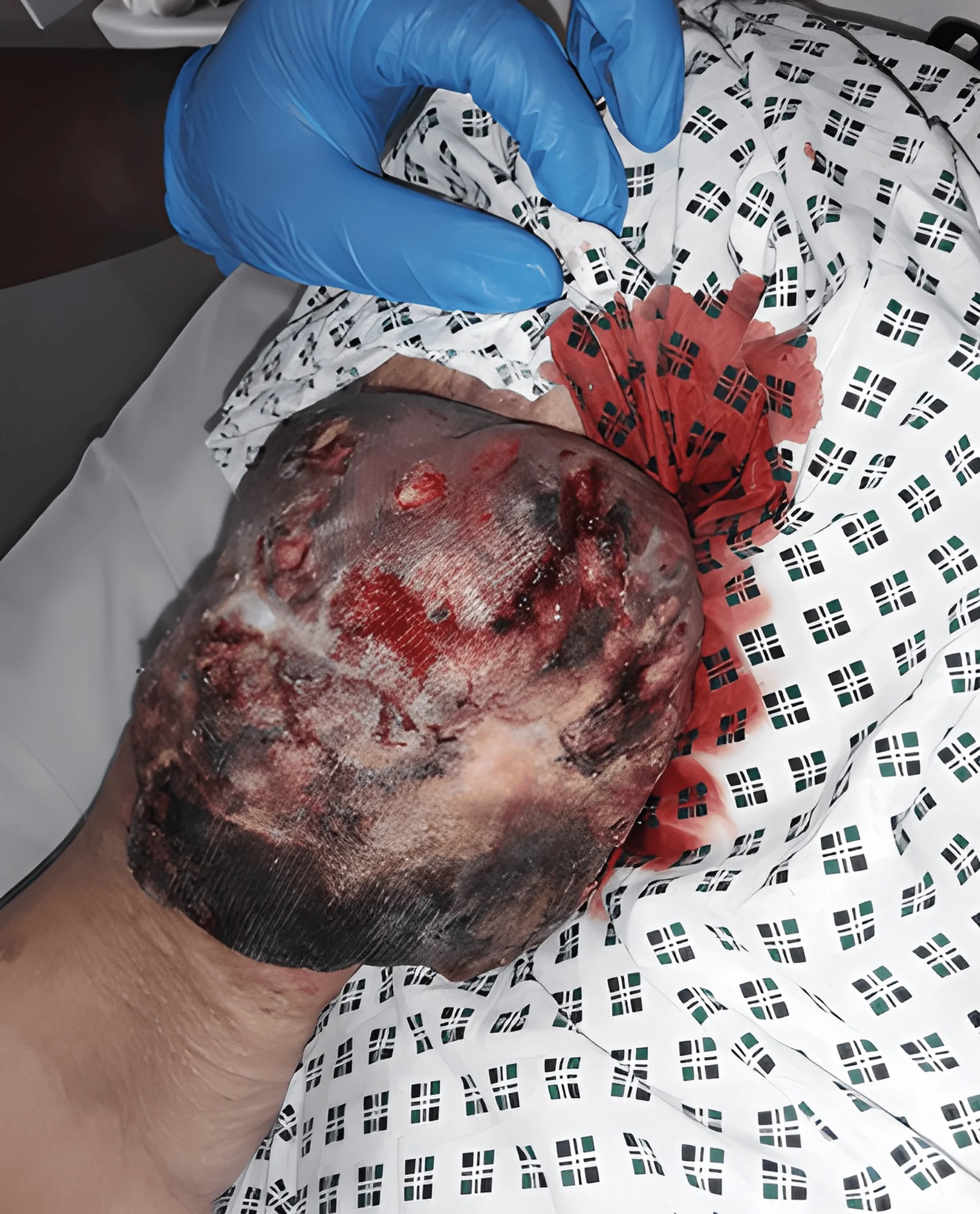
Right forearm: Advanced ulcerated lesion with an irregular necrotic surface and visible bleeding.

**Figure 4:**
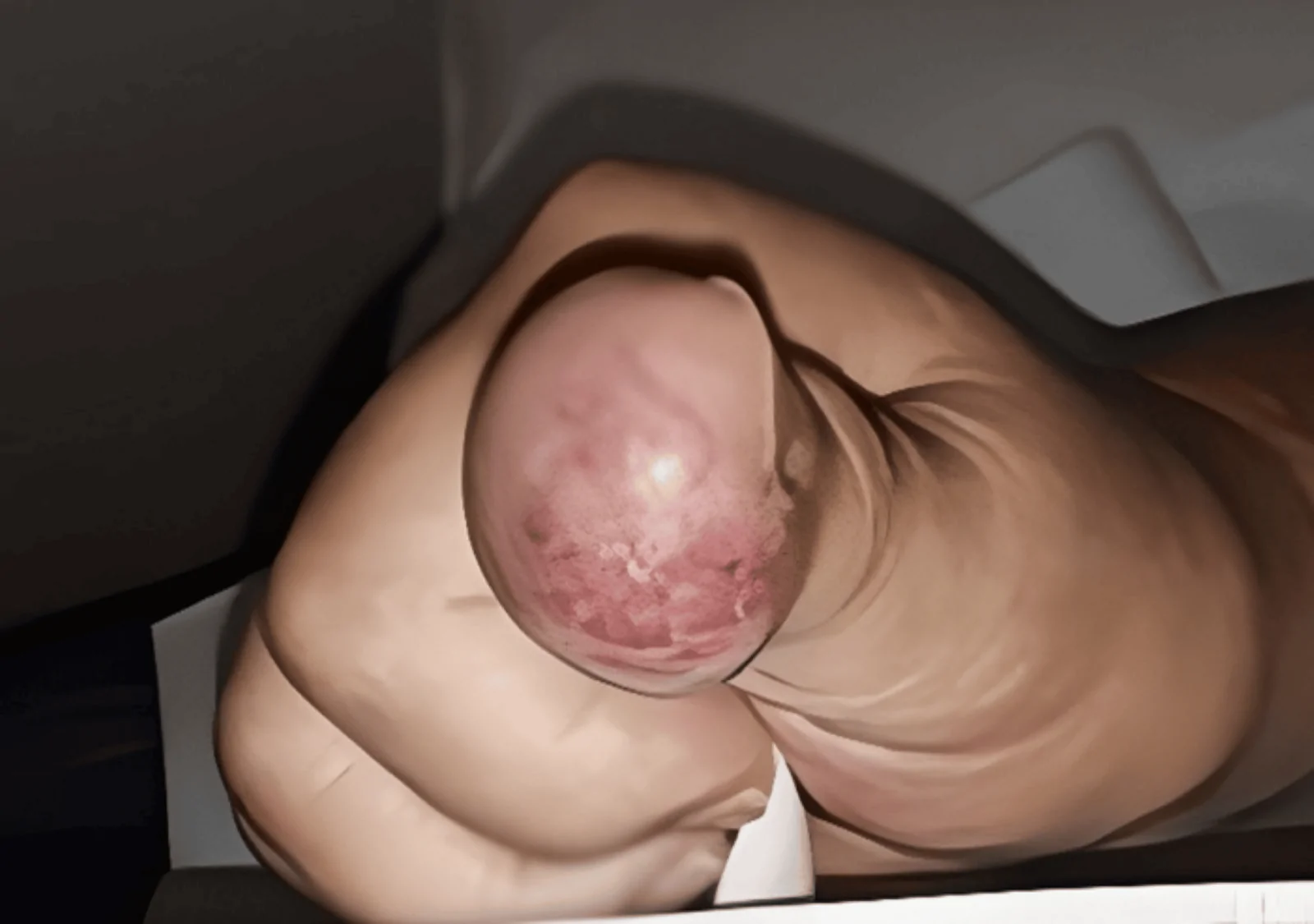
Right thumb: Early rounded nodular lesion before extensive ulceration.

**Figure 5:**
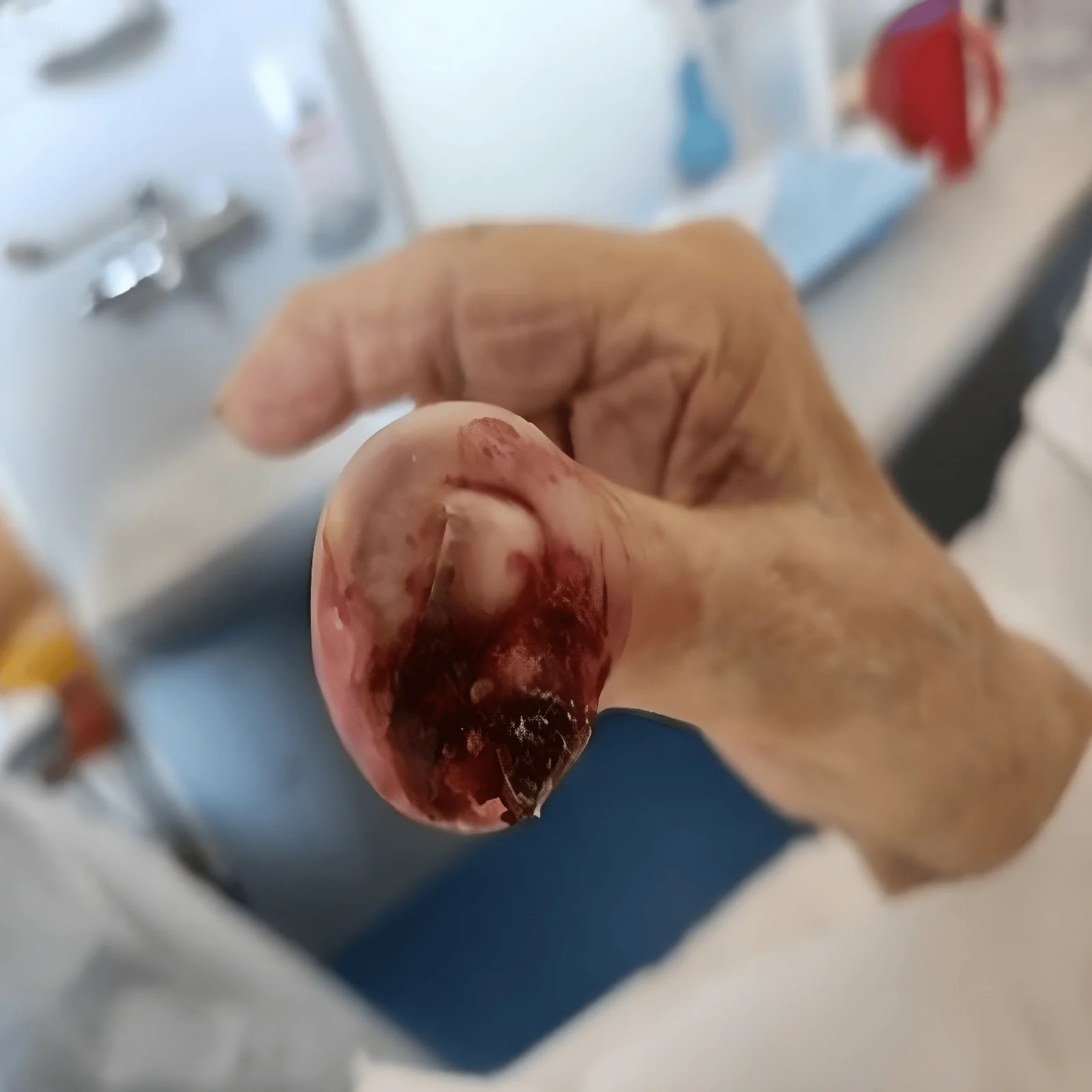
Right thumb: Subsequent ulceration and hemorrhagic changes involving the distal thumb.

**Figure 6:**
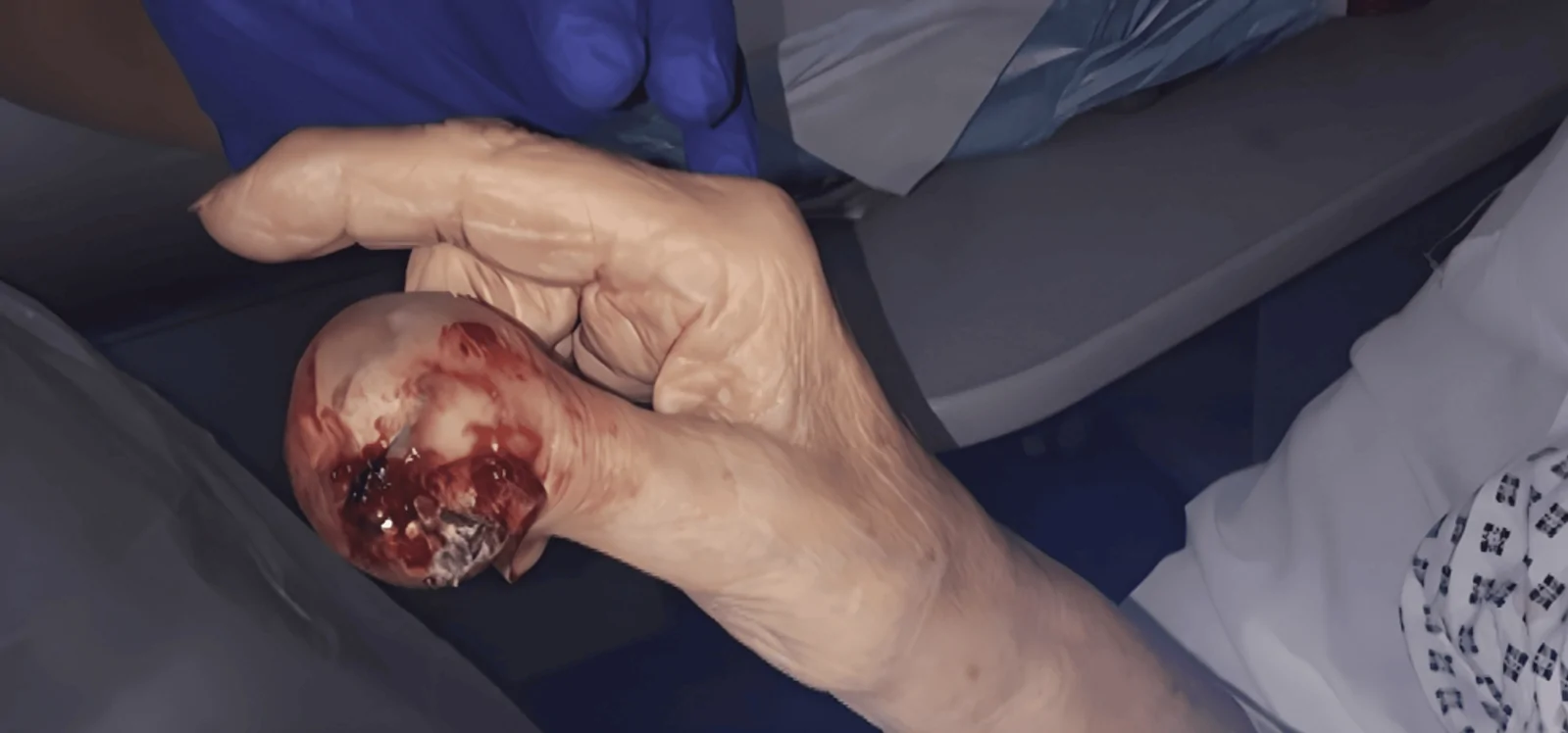
Right thumb: Further clinical view of the ulcerated distal lesion.

Around the same time, the patient experienced an accidental fall and presented to the Accident and Emergency Department, where both lesions were clinically assessed. Subsequent investigations, including imaging and biopsy, confirmed that the lesions represented cutaneous metastases originating from a primary lung carcinoma [5]. MRI of the skin lesions demonstrated soft-tissue lesions corresponding to the clinically apparent forearm and thumb abnormalities (Figures 7-9) [6].

**Figure 7:**
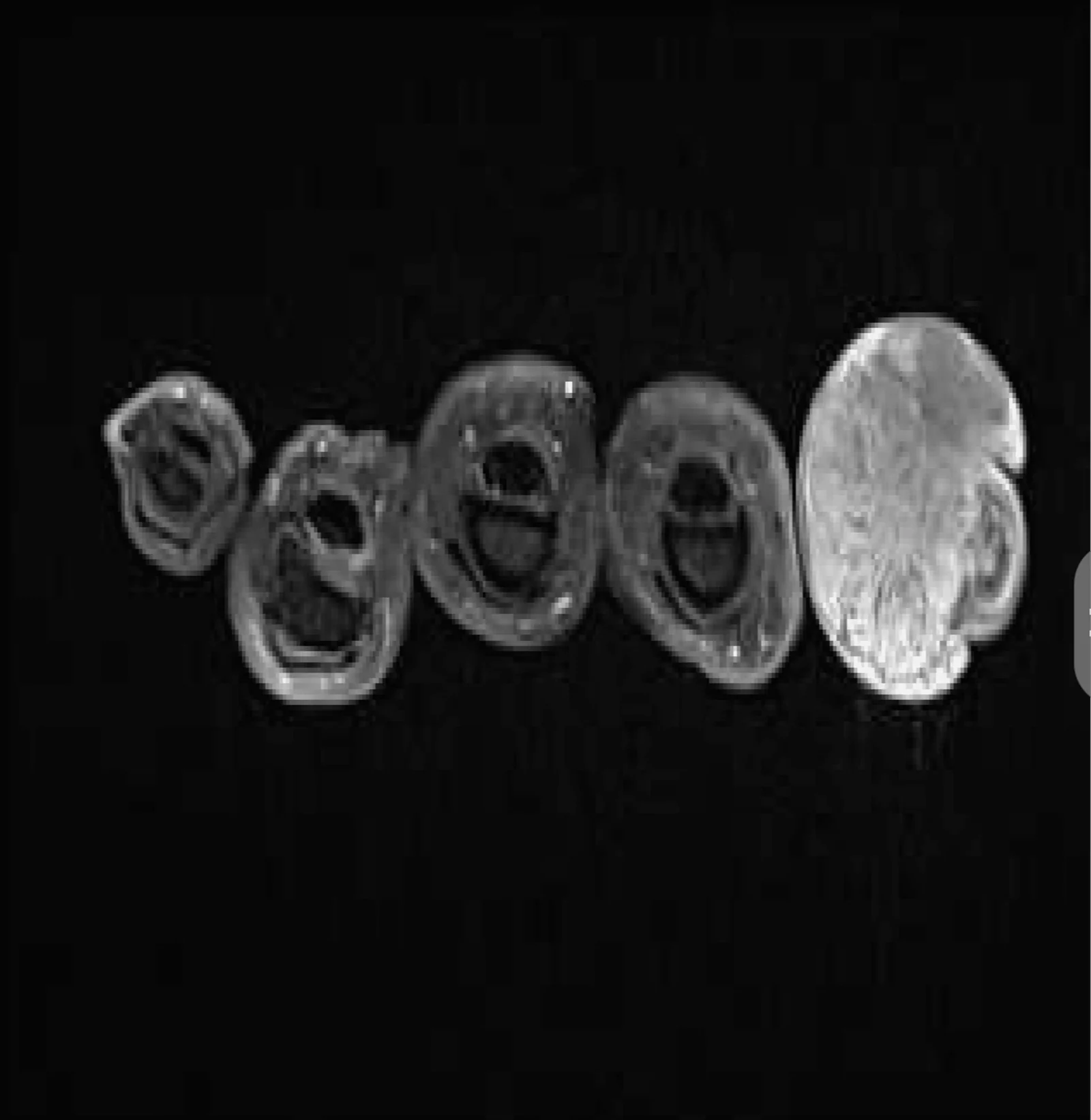
MRI of the hand: Axial image demonstrating a large soft-tissue lesion involving the thumb.

**Figure 8:**
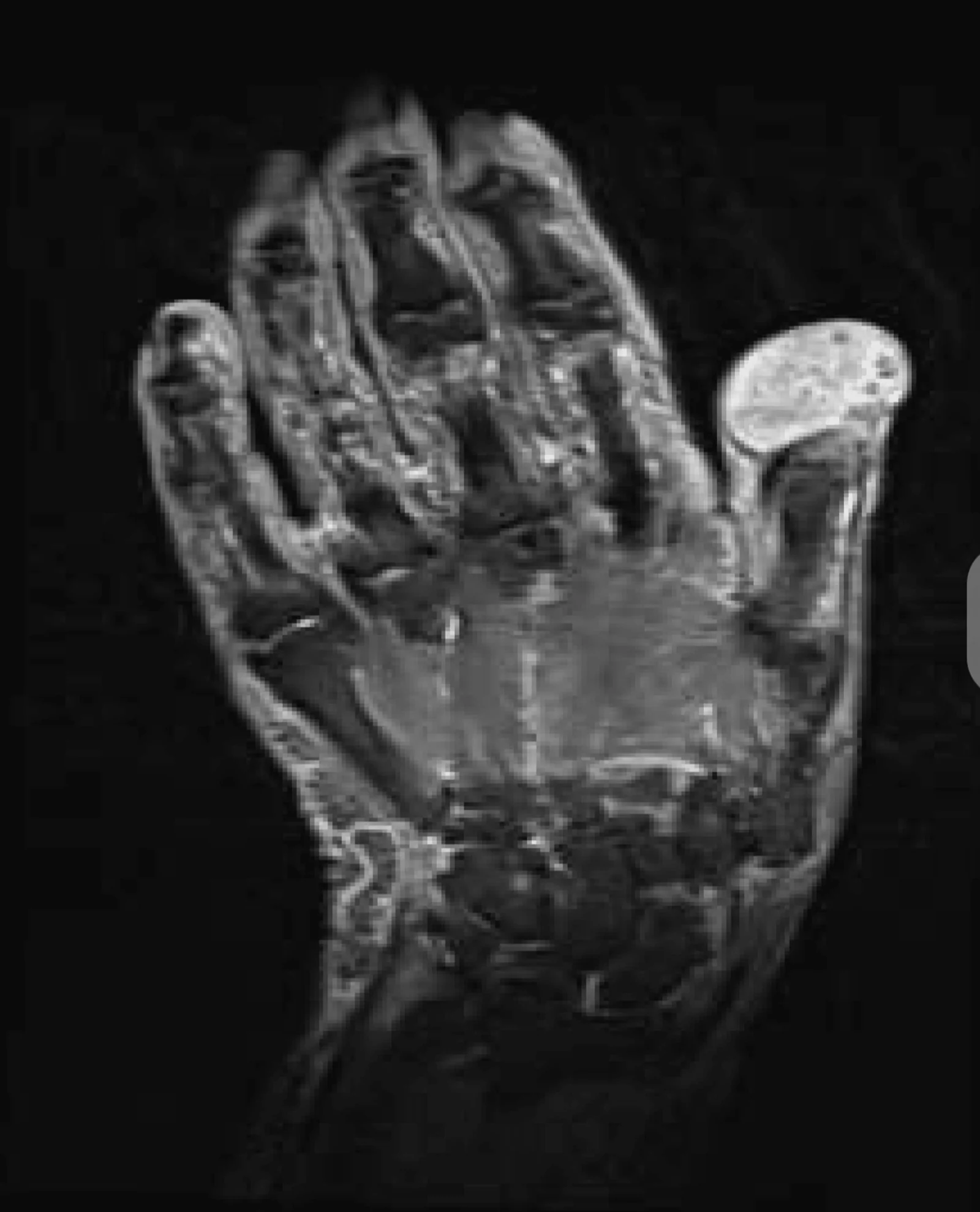
MRI of the hand: Coronal image demonstrating the thumb soft-tissue lesion.

**Figure 9:**
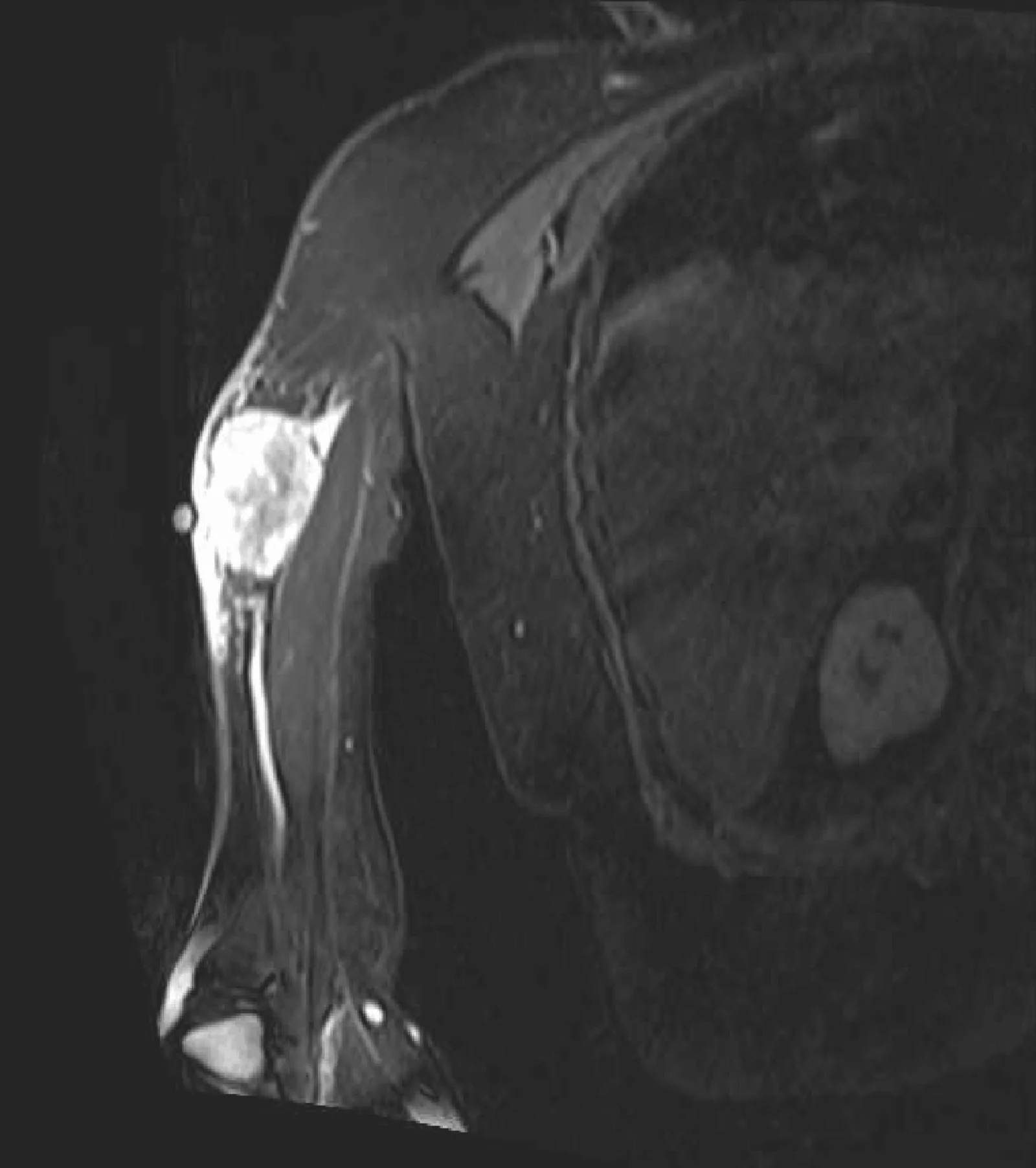
MRI of the right upper limb: Soft-tissue lesion at the distal upper arm/forearm region shown in the submitted image.

The patient had a WHO performance status of 3 and a clinical frailty score of 3. Her smoking history was significant, with an estimated 80 pack-years. She stopped active smoking 20 years ago and had stopped vaping five weeks before this admission. She denied any systemic symptoms and remained independent and generally well.

Further diagnostic workup included a whole-body CT scan, bronchoscopy, and tissue biopsy. CT of the thorax, abdomen, and pelvis with contrast showed a 6 cm right middle lobe mass causing obstructive middle lobe atelectasis and a right apical bronchocele. A few sub-3 mm faceted subpleural upper-lobe nodules were interpreted as intrapulmonary lymph nodes. There was no significant supraclavicular or mediastinal lymphadenopathy (Figure 10).

**Figure 10:**
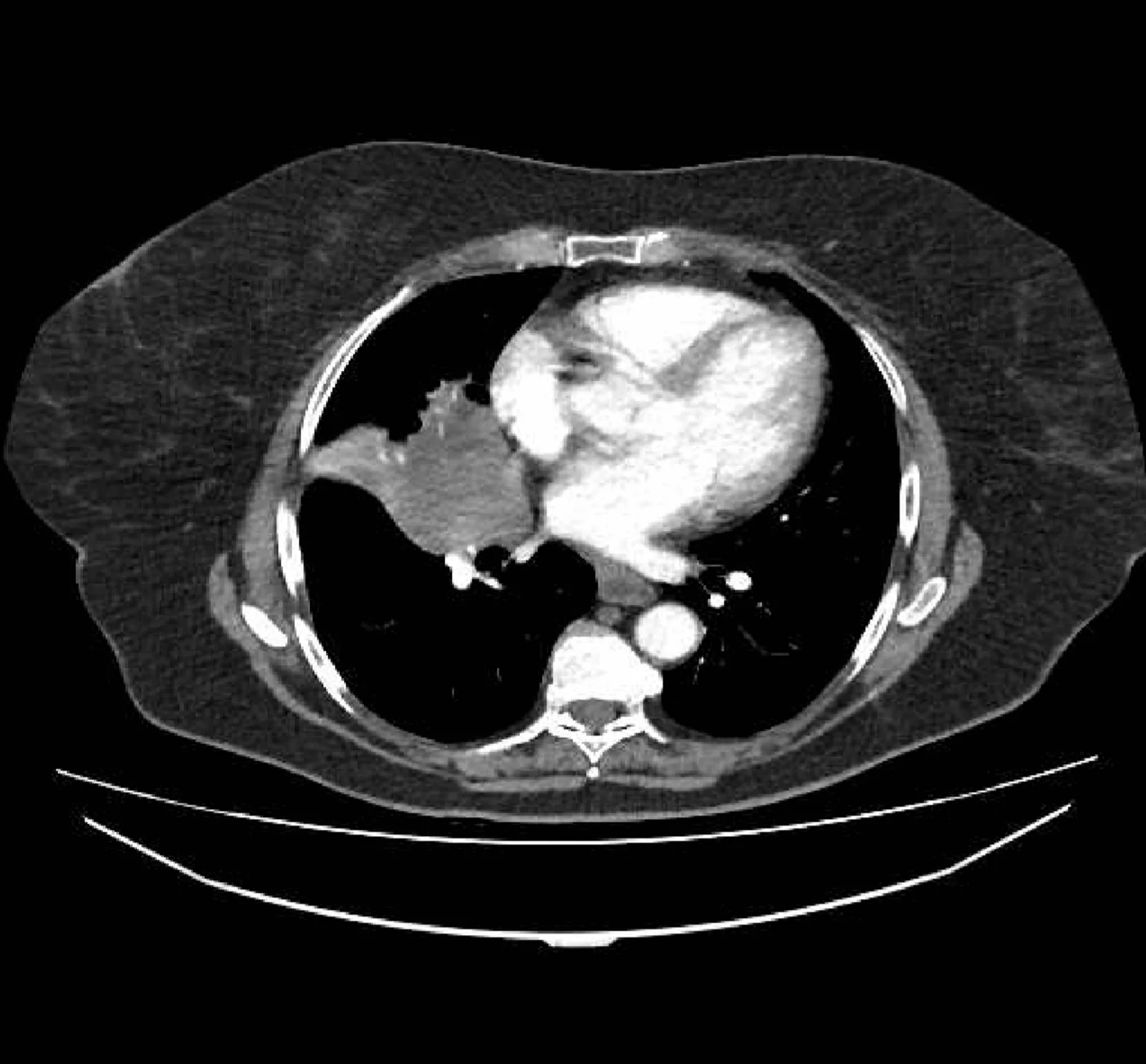
Contrast-enhanced axial CT of the chest showing a right-sided pulmonary mass.

Two 18-gauge core biopsies were obtained from the presumed subcutaneous component of the right arm mass. Histology showed fibrous tissue extensively infiltrated by a high-grade malignant tumor forming nests and islands of basaloid cells with hyperchromatic nuclei, scant cytoplasm, extensive central comedo-type coagulative necrosis, numerous atypical mitoses, and focal cytoplasmic and extracellular keratinization; focal rosette-like structures were suggested.

Immunohistochemistry was strongly and diffusely positive for p16 and BerEP4, strongly positive for MOC31 and p53, and showed extensive but patchy CK5/6 and EMA staining with a reciprocal distribution. CK7 stained patchily in more squamous-appearing areas, and CEA showed heterogeneous/biphasic staining. Tumor cells were negative for p63, androgen receptor, CD56, and CK20. The MIB-1 proliferation index exceeded 90%. The pathologist considered a very poorly differentiated carcinoma with both squamous and basaloid/adnexal differentiation; metastasis from a noncutaneous primary and a primary cutaneous adnexal tumor were both considered. CD56 and CK20 negativity argued against Merkel cell carcinoma.

Separately, biopsy of the right middle lobe mass showed partially necrotic and hemorrhagic bronchial/stromal tissue infiltrated by atypical basaloid nests with focal possible intercellular bridges and keratinization. The pulmonary tumor was CK5/6-positive, p40-negative, and p63-negative, with an MIB-1 index of >90%; the pathological conclusion was poorly differentiated basaloid squamous cell carcinoma. Although p40 and p63 were negative in the lung biopsy, the diagnosis was based on the overall morphology and immunophenotype rather than these markers in isolation. Concordant basaloid/squamous features in the two specimens, together with the imaging and clinical context, supported a pulmonary primary with a right arm metastasis.

At the multidisciplinary team meeting, the recorded clinical stage was T4 N2 M1c, and the most likely primary site was the lung, with a soft-tissue metastasis to the right arm. Performance status was documented as 3, and the team considered the patient unlikely to benefit from systemic anticancer therapy (SACT) [7]. Palliative radiotherapy was subsequently delivered to the right arm lesion over several treatment sessions for symptomatic bleeding. Tissue-viability nurses provided specialist care for the malignant forearm wound, focusing on atraumatic dressings, control of exudate and malodor, and symptom relief.

Wound dressings for rapidly growing necrotic tumors differ from standard dressings for necrotic wounds. The former focus on symptom relief, reducing exudate, and pain relief, whereas the latter aim at debridement and facilitating tissue regeneration. A nontraumatic, odor-controlling dressing such as Cinesteam is used to eliminate unpleasant odors and absorb exudate. It consists of an absorbent layer together with a sachet containing a natural spice fragrance (e.g., cinnamon). It allows the passage of air and moisture to prevent maceration. It is used for necrotic tumors, whereas hydrocolloid or alginate dressings are used for necrotic ulcers [8].

## Discussion

Non-small cell lung cancer (NSCLC) accounts for 80-85% of all lung cancers and includes squamous cell carcinoma, large cell carcinoma, and adenocarcinoma. There are sparse clinical data on NSCLC metastasizing to unusual sites in the literature. The Global Burden of Diseases, Injuries, and Risk Factors Study 2023, as published in The Lancet, reports that approximately 40-50% of lung cancer cases are metastatic at initial diagnosis, with the brain, bones, liver, and adrenal glands being the most frequently affected sites [1,2].

This case illustrates an uncommon presentation of metastatic lung squamous cell carcinoma through progressive lesions of the forearm and thumb. Skin involvement may be the first clinically recognized sign of lung cancer, even when respiratory or constitutional symptoms are not reported [3-5]. The distal distribution is particularly noteworthy because published case series more often describe lesions on the chest wall, abdomen, head, or neck. A cutaneous nodule that enlarges quickly, ulcerates, or bleeds should prompt reassessment of the differential diagnosis and consideration of biopsy.

The key differential diagnoses include primary cutaneous squamous cell carcinoma, keratoacanthoma-type squamous proliferation, and squamous malignancy arising in a chronic scar or wound (Marjolin ulcer), as well as adnexal tumors, infection, and other metastatic malignancies. Clinical appearance alone is insufficient to distinguish these entities. Histological assessment for epidermal connection or in situ change, comparison with a known internal tumor, and a carefully selected immunohistochemical panel may help. Markers such as p40, p63, and CK5/6 support squamous differentiation but are not, by themselves, specific for pulmonary versus cutaneous origin; TTF-1 is commonly negative in pulmonary squamous carcinoma. Therefore, no single stain proves the origin in every case. In this patient, concordant poorly differentiated squamous morphology in the skin and lung specimens, together with the CT-detected lung mass and multidisciplinary team review, supported metastatic pulmonary disease.

Cutaneous metastasis from lung cancer generally indicates advanced malignancy and has been associated with short survival in retrospective series, although individual outcomes vary and cannot be predicted from this case alone [7]. Treatment should be tailored to tumor biology, stage, performance status, comorbidity, patient preference, and symptom burden. Depending on clinical circumstances, options may include SACT and local measures such as palliative radiotherapy for bleeding, pain, or troublesome tumor bulk.

Malignant wounds require a symptom-oriented rather than routinely debriding approach. Practical priorities include atraumatic dressing changes, management of exudate and odor, hemostasis when needed, analgesia, and psychosocial support. Specialist tissue-viability input in this case illustrates the contribution of multidisciplinary palliative care alongside oncological assessment.

## Conclusions

Cutaneous metastases from internal malignancies are rare, particularly in the absence of systemic symptoms, and often present diagnostic challenges. Rapidly progressive, ulcerating lesions on the forearm or thumb may rarely be the presenting manifestation of metastatic lung carcinoma, even without reported systemic symptoms. Early biopsy and correlation of skin histology with cross-sectional imaging and tissue from a suspected primary site are essential to distinguish metastatic disease from a primary cutaneous malignancy. Multidisciplinary evaluation helps establish an appropriate diagnosis and patient-centered management plan, including specialist palliative wound care. This single case does not establish that earlier recognition improves survival. This case underscores the importance of recognizing unusual metastatic patterns of NSCLC, as early identification of cutaneous lesions may prompt timely evaluation for systemic progression and facilitate multidisciplinary therapeutic decision-making.
